# Simultaneous tracking of fly movement and gene expression using GFP

**DOI:** 10.1186/1472-6750-8-93

**Published:** 2008-12-16

**Authors:** Dhruv Grover, Junsheng Yang, Simon Tavaré, John Tower

**Affiliations:** 1 Molecular and Computational Biology Program, Department of Biological Sciences, University of Southern California, Los Angeles, CA 90089-2910, USA; 2 Department of Oncology, University of Cambridge, Cambridge, CB2 2XZ, UK

## Abstract

**Background:**

Green Fluorescent Protein (GFP) is used extensively as a reporter for transgene expression in Drosophila and other organisms. However, GFP has not generally been used as a reporter for circadian patterns of gene expression, and it has not previously been possible to correlate patterns of reporter expression with 3D movement and behavior of transgenic animals.

**Results:**

We present a video tracking system that allows tissue-specific GFP expression to be quantified and correlated with 3D animal movement in real time. *eyeless/Pax6 *reporter expression had a 12 hr period that correlated with fly activity levels.

*hsp70 *and *hsp22 *gene reporters were induced during fly aging in circadian patterns (24 hr and 18 hr periods, respectively), and spiked in the hours preceding and overlapping the death of the animal. The phase of *hsp *gene reporter expression relative to fly activity levels was different for each fly, and remained the same throughout the life span.

**Conclusion:**

These experiments demonstrate that GFP can readily be used to assay longitudinally fly movement and tissue-specific patterns of gene expression. The *hsp22*-GFP and *hsp70*-GFP expression patterns were found to reflect accurately the endogenous gene expression patterns, including induction during aging and circadian periodicity. The combination of these new tracking methods with the *hsp*-GFP reporters revealed additional information, including a spike in *hsp22 *and *hsp70 *reporter expression preceding death, and an intriguing fly-to-fly variability in the phase of *hsp70 *and *hsp22 *reporter expression patterns. These methods allow specific temporal patterns of gene expression to be correlated with temporal patterns of animal activity, behavior and mortality.

## Background

The green fluorescent protein (GFP) gene isolated from the jellyfish *Aequorea victoria *encodes a protein that absorbs blue light and emits green light. GFP and related fluorescent proteins [1] have been used extensively as reporter molecules for transgene expression in a variety of cells and transgenic animals, including models for the study of behavior such as *Drosophila melanogaster*, zebrafish and mouse [2-4]. Video tracking of Drosophila [5-7] and other animals [8-10] is increasingly being used in studies of movement and behavior. Methods to correlate efficiently such movements and behaviors with levels of, and changes in, gene expression would potentially provide insight into regulatory pathways [11]. Aging is a research area where longitudinal and simultaneous assay of animal movement and gene expression might be particularly informative: Aging in Drosophila and other organisms results in deterioration of movements and behaviors [12-15], and aging is associated with characteristic changes in gene expression patterns [16-22]. We present a real-time image acquisition system that allows quantification of GFP fluorescence intensity as a read-out of gene expression and the simultaneous tracking of 3D animal movement and behavior. These methods allow specific temporal patterns of gene expression to be correlated with temporal patterns of animal activity, behavior, and mortality.

## Results and discussion

Transgenic fly strains were utilized in which enhanced-fluorescence GFP (eGFP) [23] is expressed specifically in retina tissue in adults by an artificial promoter (called *3xP3*) containing three binding sites for the eyeless/PAX6 homeodomain transcription factor [24]. eGFP was also expressed in adult flies using reporters containing the promoters of the stress-response genes *hsp70 *and *hsp22*, which are induced in tissue-specific patterns during aging [16,17,25,26]. The construction and characterization of the *hsp70*-GFP and *hsp22*-GFP reporters will be described in detail elsewhere (JY and JT, submitted). We have previously described a real-time image acquisition system that allows tracking of fly movement through 3D space using visible light [27]. Multiple video cameras were used to obtain fly silhouettes and construct three-dimensional visual hulls of each fly, and an Extended Kalman Filter algorithm was used to estimate reliably the location of each fly based on past positions, allowing the tracking of fly movement at 60 frames/sec. To allow detection of GFP fluorescence, inexpensive blue LED lights were used to illuminate the flies, and colored filters were placed over the camera lenses to filter out reflected blue light and allow GFP fluorescence to pass through (Methods); in the absence of the filters the reflected blue light would be so bright as to preclude detection of the GFP fluorescence.

A tracking algorithm was developed to detect GFP fluorescence in the camera images and track the GFP-expressing flies in 3D at a frame rate of 60 frames/sec. The spatial position of the fly was determined by sampling points on the surface of the visual hull and computing the centroid. Since GFP is expressed in both eyes of the *3xP3-GFP *flies, this resulted in two visual hulls or clusters of points. For these flies the center point of the two clusters was used as the 3D spatial position of the fly. Activity values were generated by summing the distance traveled in cm by the fly in a specific time interval (cm/sec or cm/hr depending on the experiment). The intensity of the fluorescence was calculated for each frame by averaging the intensity of each green fluorescent pixel in the camera images, and the average GFP intensity for the time interval is presented; detailed protocols are available for download from the laboratory website .

**Figure 1:**
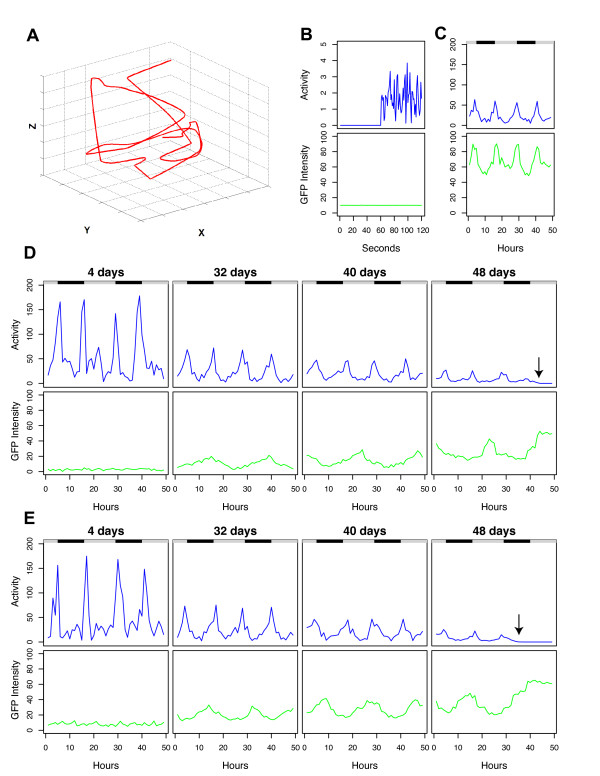
**Tracking fly movement and gene expression using GFP**. (A) A young *3xP3*-GFP male fly was tracked for 120 seconds using GFP, and the 3D trajectory is indicated in red. (B) A male *hsp70*-GFP fly (40 days old, strain 2MI4) was tracked for 120 seconds, flanking a transition from motionless to sustained activity. Activity is expressed as distance moved per second (blue) and GFP intensity is expressed as average pixel intensity per second (green). (C-E) Single flies of the indicated age were tracked for 48 hours. Black/white bars indicate the light/dark cycle the flies were cultured under prior to the beginning of the experiment. Activity is expressed as distance moved per hour (blue) and GFP intensity is expressed as average pixel intensity per hour (green). (C) *3xP3*-GFP, transgenic strain M1, 37 days old. (D) *hsp70*-GFP, transgenic strain 2MI4. (E) *hsp22*-GFP, transgenic strain 1MI1. Arrows indicate the last time point at which the 48-day-old animals displayed spontaneous movement; these animals were scored as dead at the end of the experiment based on lack of movement in response to stimulation.

The GFP tracking system was tested using transgenic flies containing one of three transgenic reporter constructs, *3xP3*-GFP, *hsp70*-GFP or *hsp22*-GFP. Results were confirmed using multiple independent transgenic lines for each reporter construct (Table 1). Individual flies in a culture vial were placed in the camera rig in a dark room and bathed with blue light from the LED. GFP fluorescence readily allowed reliable and uninterrupted tracking of the fly trajectories through 3D space (Figure 1A), coincident with quantification of GFP fluorescence intensity. The sensitivity of the assay will depend upon the amount of expression as well as the tissue in which the expression occurs. We observed that even GFP fluorescence from internal tissues that was dim and difficult to detect by eye using a fluorescence stereomicroscope was readily assayed using the video camera system, for example the young *hsp*-GFP flies described below. Controls demonstrated that fly movement does not interfere with assay of GFP intensity: flies were tracked when they transitioned from stationary to actively moving, and this did not alter the level of GFP intensity detected (Figure 1B; and additional data not shown). To allow correlation of gene expression patterns with circadian fly activity patterns, an adult male fly containing the *3xP3*-GFP reporter was tracked for 48 hours using GFP fluorescence (Figure 1C). The expected "crepuscular" circadian activity pattern was detected, with peaks of fly movement activity occurring approximately every 12 hours, corresponding to the dusk and dawn of the previous culture conditions (indicated with light/dark bars). Expression of the *3xP3*-GFP reporter was found to vary dramatically over the course of the 48 hour experiment. Regular peaks of expression level were detected with a periodicity of ~12 hours that corresponded closely to the ~12-hour activity period (Figure 1C). Circadian expression or activity has not been previously reported for Drosophila *eyeless*, however the mammalian homolog *Pax6 *exhibits robust circadian expression in adult animals [28].

**Table 1:** Summary of activity and GFP intensity data for tracked flies.

**Genotype**	**Age**	**Sex**	**Activity**		**GFP Intensity**		**Death**
			**Mean**	**Period**	**Mean**	**Period**	
*eyeless unknown*	unknown	Male	28.37	12	63.9	11.67	
*eyeless I4*	37	Male	22.71	12	56.97	12.33	
*eyeless M1*	37	Male	22.53	12	66.63	12.67	
							
*hsp70 GFP(3) 2MI4/TM3 (a)*	4	Male	52.75	12	3.96	N/A	
*hsp70 GFP(3) 2MI4/TM3*	4	Male	60.26	12	4.06	N/A	
*hsp70 GFP(3) 1MI2/TM3*	4	Male	48.51	11.67	4.18	N/A	
*hsp70 GFP(3) 2MI4/TM3 (a)*	32	Male	23.24	12	10.66	24	
*hsp70 GFP(3) 2MI4/TM3 (a)*	40	Male	27.48	12	15.88	24	
*hsp70 GFP(3) 2MI4/TM3*	40	Male	19.82	12	14.46	23	
*hsp70 GFP(3) 2MI4/TM3 (a)*	46	Male	15.87	12	22.18	24	
*hsp70 GFP(3) 2MI4/TM3*	48	Female	7.43	12	27.41	23	X
*hsp70 GFP(3) 2MI4/TM3 (a)*	53	Male	13	12	24.28	24	
							
*hsp22 GFP(3) 1MI1/TM3 (b)*	4	Male	46.16	12	8.14	N/A	
*hsp22 GFP(3) 1MI1/TM3*	4	Male	64.77	11.67	8.22	N/A	
*hsp22 GFP(2) 1 [homozygous]*	4	Male	52.56	12	8.9	N/A	
*hsp22 GFP(3) 1MI1/TM3 (b)*	32	Male	24.01	12	20.18	18	
*hsp22 GFP(3) 1MI1/TM3 (b)*	40	Male	23.62	12	26.91	18	
*hsp22 GFP(3) 1MI1/TM3*	40	Male	20.32	12	27.6	18.5	
*hsp22 GFP(3) 1MI1/TM3 (b)*	46	Male	8.05	12	38.23	18	X
*hsp22 GFP(3) 1MI1/TM3*	48	Male	5.8	12	40.37	18	X

All transgenic constructs are marked with *white*+ and are in a *white *[1118] genetic background. Flies that died during the course of the 48-hour tracking experiment are indicated with an X. (a) and (b) indicates data generated from the same fly at different ages. Flies subjected to starvation/dessication are not included.

The expression of the *hsp70*-GFP and *hsp22*-GFP reporters could also be assayed longitudinally and correlated with fly behavior and mortality. Both *hsp70 *and *hsp22 *genes increase in expression during aging of adult flies [16,17,25]. Ceriani and coworkers [29] have used microarrays to assay genome-wide message levels in adult flies, and report that *hsp22 *is circadian-regulated, with a period of 17.6 hours, and that an *hsp70*-related gene is circadian-regulated with a period of 23.78 hours. Adult male flies of increasing age, containing either the *hsp70*-GFP reporter or *hsp22*-GFP reporter, were tracked for periods of 48 hours to reveal circadian patterns of fly activity, and allow correlation with GFP expression level. As expected fly movement decreased in amount and rhythmicity during aging (Figure 1D, E) [13]. The expression of *hsp70*-GFP and *hsp22*-GFP transgenes was low in young animals and induced in old animals, as expected. Strikingly, both *hsp70*-GFP expression (Figure 1D) and *hsp22*-GFP expression (Figure 1E) was circadian-regulated, with periods of ~24 hours and ~18 hours, respectively, and therefore in good agreement with the previously reported circadian variation in *hsp *message levels. In addition, expression of both *hsp70*-GFP and *hsp22*-GFP transgenes was observed to spike to their highest levels in the hours preceding and overlapping the death of the animal (Figure 1D, E). Aging in Drosophila and other organisms correlates with increased oxidative stress, and this oxidative stress is causally implicated in the induction of *hsp70 *and *hsp22 *genes during aging [26]. The rhythmic patterns of *hsp *reporter expression observed here suggest that the oxidative stress associated with aging may also be rhythmic and spike immediately prior to death.

**Figure 2:**
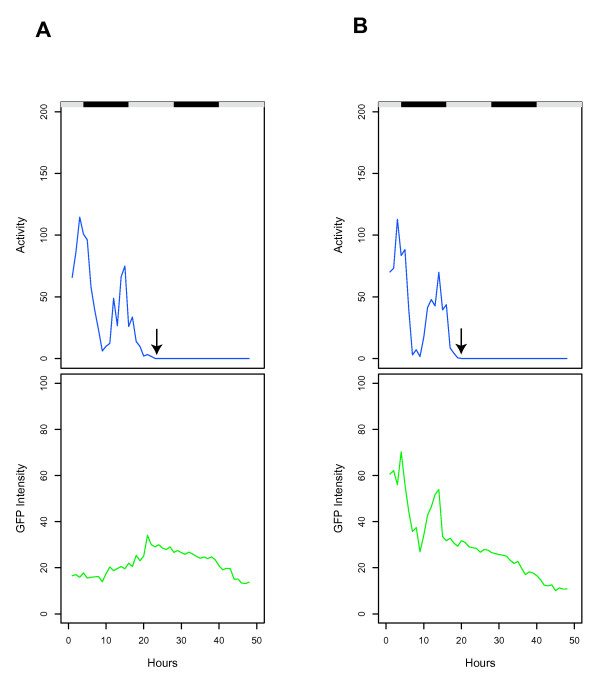
**GFP reporter expression in flies dying from dessication/starvation**. Single male flies were placed in empty culture vials and tracked for 48 hours. Black/white bars indicate the light/dark cycle the flies were cultured under prior to the beginning of the experiment. Activity is expressed as distance moved per hour (blue) and GFP intensity is expressed as average pixel intensity per hour (green). Arrows indicate the last time point at which the animals displayed spontaneous movement; these animals were scored as dead at the end of the experiment based on lack of movement in response to stimulation. (A) *hsp22*-GFP, transgenic strain 1MI1. (B) *3xP3*-GFP, transgenic strain I4. The experiment was repeated with similar results.

To determine if a spike in GFP reporter expression would be observed when flies died due to an acute stress, the *hsp22*-GFP reporter flies and the *3xP3*-GFP controls were placed in empty culture vials and tracked for 48 hours (Figure 2). When adult flies are deprived of both water and food they will die within one or two days, due primarily to dessication [30]. GFP fluorescence was observed to spike over the ~10 hours preceding the last spontaneous movement by the *hsp22*-GFP fly (Figure 2A). In contrast, with the *3xP3*-GFP control fly, the fluorescence level closely paralleled the decreasing activity levels, and declined in the last ~5 hours preceding the last spontaneous movement of the animal (Figure 2B). These results suggest that the spike in GFP fluorescence observed with the *hsp*-GFP reporters is due to increased expression of GFP, rather than an increase in GFP stability in dying flies.

**Figure 3:**
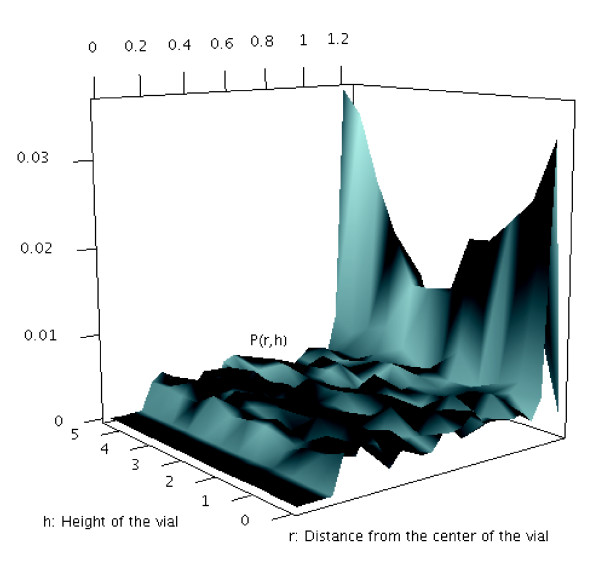
**Location of the fly in the vial and centrophobism behavior**. A single male *3xP3*-GFP fly was tracked for 48 hours, and the probability of the fly residing in a particular location relative to the center of the vial for a particular height is plotted.

Additional fly behaviors could also be assayed by tracking GFP fluorescence. For example, a single male *3xP3*-GFP fly was tracked for 48 hours, and the probability of the fly residing in a particular location relative to the center of the vial for a particular height was plotted (Figure 3). The plot reveals the tendency for the fly to explore the edges of the vial, a behavior called "centrophobism" [31], as well as a preference for the top and bottom of the vial.

**Figure 4:**
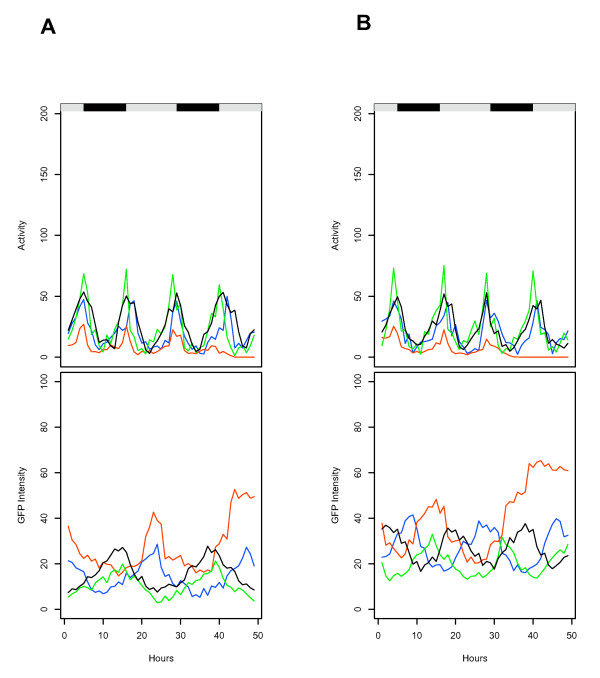
**Comparison of activity and *hsp*-GFP expression data from different flies**. The data for tracking of several different flies over a period of 48 hours is overlaid to allow comparison of fly-to-fly variability in activity (upper panel; distance moved per hour) and GFP intensity (lower panel; average pixel intensity per hour). Black/white bars indicate the light/dark cycle the flies were cultured under prior to the beginning of the experiment. Different colors indicate different individual flies, some of slightly different ages. (A) *hsp70*-GFP transgenic flies, strain 2MI4. Green, 32 days; Blue, 40 days; Black, 40 days; Red 48 days. The 48-day old fly (Red) dies in the course of the experiment. (B) *hsp22*-GFP transgenic flies, strain 1MI1. Green, 32 days; Blue, 40 days; Black 40 days; Red, 48 days. The 48-day old fly (Red) dies in the course of the experiment.

**Figure 5:**
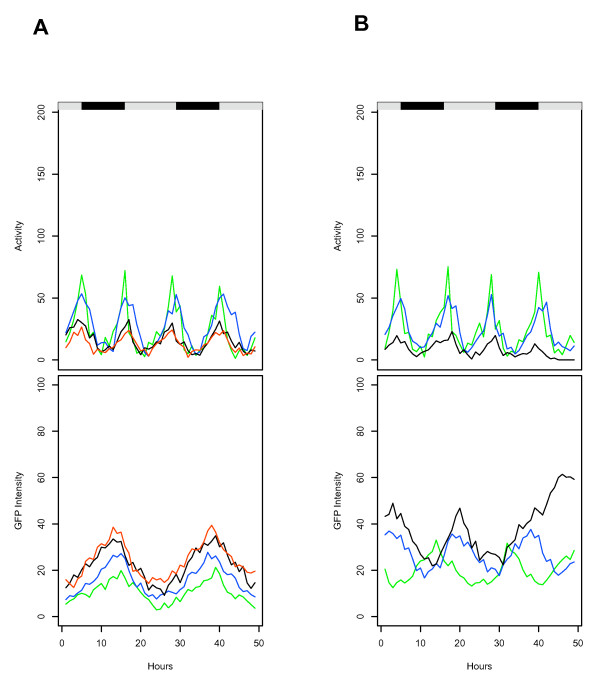
**Comparison of activity and *hsp*-GFP expression data from the same fly at different ages**. The data for tracking of the same fly over a period of 48 hours at different ages is overlaid to allow comparison of activity (upper panel; distance moved per hour) and GFP intensity (lower panel; average pixel intensity per hour). Black/white bars indicate the light/dark cycle the flies were cultured under prior to the beginning of the experiment. Different colors indicate the same fly at different ages (see Table 1). (A) *hsp70*-GFP transgenic fly, strain 2MI4, fly "a". Green, 32 days; Blue, 40 days; Black, 46 days; Red, 53 days. (B) *hsp22*-GFP transgenic fly, strain 1MI1, fly "b". Green, 32 days; Blue, 40 days; Black, 46 days. The 46-day old fly dies during the course of the experiment. Note: the overlap of the GFP intensity plots for the *hsp22*-GFP fly is not obvious because of the 18-hour period, however if this is taken into account the peaks are found to coincide.

Finally, an intriguing fly-to-fly variability in reporter expression patterns was observed: the *hsp70 *and *hsp22 *transgenic reporters consistently exhibited ~24 hour or ~18 hour periods of expression, respectively. However these periods of *hsp *reporter expression were in slightly different phases relative to fly activity level in different individual flies (Figure 4), and were maintained for the same fly at different ages (Figure 5). The reason for this variability is not clear, and it will be of interest in the future to determine if it might be related to variability in fly life spans.

## Conclusion

These experiments demonstrate that GFP can readily be used to assay longitudinally fly movement and gene expression, and that typical GFP variants such as eGFP are sufficiently short-lived to allow the detection of a variety of circadian gene expression patterns. Using GFP tracking, the *hsp22*-GFP and *hsp70*-GFP expression patterns were found to reflect accurately the endogenous gene expression patterns, including induction during aging and circadian periodicity. The combination of these new tracking methods with the *hsp*-GFP reporters revealed additional information. These newly identified aspects include a spike in *hsp22 *and *hsp70 *reporter expression in the hours preceding and overlapping the death of the animal. Moreover, there was an intriguing fly-to-fly variability in the phase of *hsp *gene reporter expression: The phase of *hsp *gene reporter expression relative to fly activity levels was different for each individual fly, and this fly-specific pattern remained the same throughout the life span of that particular individual. While the current technology allows only single flies to be tracked using GFP, we are working to develop methods that will in the future allow simultaneous tracking and assay of GFP in multiple flies, analogous to the multiple-fly tracking that is possible using visible light [27].

Previously luciferase has been used as a reporter for circadian gene expression *in vivo *in Drosophila and certain other organisms [32,33]. However the usefulness of luciferase is limited by the requirement for constant feeding of the substrate luciferin, which has unknown consequences for phenotypes such as stress resistance and life span. Moreover, the number of luciferase transgenic strains is limited. In contrast, a large number of strains exist where GFP or some other auto-fluorescent protein is used as a reporter for specific gene expression in Drosophila and other organisms. While we have not yet tested the system with other colors of fluorescent proteins, the methods should be readily adaptable to such reagents, and plans are underway to attempt tracking with dsRED. The methods presented here should facilitate the use of these rich resources in studies of behavior, circadian gene expression and aging, and allow for detailed analyses of how patterns of gene expression correlate with specific behaviors and life stages.

## Methods

### GFP tracking

Tracking of flies using GFP was accomplished by modifying procedures recently developed for tracking flies using visible light [27]. Individual flies were placed in standard 25 × 75 mm polyethylene culture vials containing 5 ml of solid food at the bottom and stoppered with cotton at the top. For certain experiments flies were placed in an empty vial to cause death by dessication/starvation. Standard 25 × 75 mm Drosophila polyethylene culture vials (Gennesee Scientific) were used for all GFP tracking experiments. The vial was placed in the center of the circular camera rig, 70 cm in diameter. Four calibrated and synchronized Flea digital cameras (Point Grey) were mounted on the camera rig, facing downwards at a distance of 15 cm from the vial. Each camera was fitted with an 8 mm megapixel fixed focal lens (Edmund Optics). To detect GFP expression in flies, the camera setup was modified with a blue-wavelength excitation light source and a long-pass visible range barrier filter. Methods were adapted from a protocol for modifying visible-light dissecting stereomicroscopes for GFP (Ian D. Chin-Sang, personal communication; ). The light source was a 5 W Luxeon V star 450 nm endura bright royal blue lambertian LED (Lumileds, Optotech, Cat # OT16-5100-RB). The LED was powered with a xitanium 700 mA LED driver (Quadica Developments, Optotech, AC converter Cat # OTMI-0060). A barrier filter was placed between each camera sensor and lens to detect GFP expression. This filter was a long pass 515 nm flexible filter (#12 straw) from a color book of 200 filters (Edmund Optics, Cat # NT39-417). In order to reduce the glare from the background when using the LED light source, a plastic translucent blue filter was placed in front of the LED. This was the blue filter from the same color book (#4290 CalColor 90). Prior to GFP tracking assay, flies were cultured to the indicated age by transfer to new food every other day, under a 13 hr/11 hr light/dark cycle, with the exception of the flies that were subjected to dessication/starvation, which were on a 12 hr/12 hr light/dark cycle. GFP tracking was conducted in a dark room where the only source of illumination was the blue LED, and all experiments were initiated at the same time of day (4 PM). Detailed protocols are available for download from the laboratory website .

### Periodicity and ARIMA modeling

The periodicity in the activity and GFP intensity data is estimated by using the Discrete Fourier Transform, a process that transforms the data into the frequency domain. We used the functions *spectrum *and *fft *in the package *stats *in R [34]. Looking at the frequency components gives us the period, since period is the inverse of the frequency. Once the periodicity in the data is obtained, an *ARIMA (p, d, q)s *process can be used to derive statistics like the mean. We chose the ARIMA model using Akaike's Information Criterion (AIC): the model with the smallest AIC was selected. Fitting was performed using the function *arima *in the package *stats *in R. The model used here included both differencing and a seasonal AR term of the form *ARIMA (1, 0, 1)s *where *s *refers to the periodicity. The fit of the model was verified via both residual analysis, the auto-correlation function of the residuals, and the portmanteau lack-of-fit test (Ljung-Box test). Results are summarized for each tracked fly in Table 1.

## Authors' contributions

JY generated and characterized transgenic fly strains, DG devised GFP tracking methods and algorithms and conducted all tracking experiments, DG and ST designed and conducted statistical analysis, JT designed the overall study, and DG and JT wrote the paper.
